# MicroRNA-23a regulates epithelial-to-mesenchymal transition in endometrial endometrioid adenocarcinoma by targeting SMAD3

**DOI:** 10.1186/s12935-016-0342-1

**Published:** 2016-09-05

**Authors:** Ping Liu, Chao Wang, Chengbin Ma, Qiongwei Wu, Wenying Zhang, Guoying Lao

**Affiliations:** Gynecology Department, Changning Maternity and Infant Health Hospital, No. 773, Wuyi Road, Changning District, Shanghai, 200051 China

**Keywords:** Endometrial endometrioid adenocarcinoma, MicroRNA, Epithelial-to-mesenchymal transition, SMAD3

## Abstract

**Background:**

To investigate the role of total cellular microRNA (miRNA) in regulating epithelial-to-mesenchymal transition (EMT) during human endometrial endometrioid adenocarcinoma (EEC).

**Methods:**

A miRCURY LNA microRNA array was used to evaluate the miRNA profiles of human EEC tissues and corresponding nontumorous endometriums. An in vitro model of TGF-β induced EMT in HEC-1-A cells was used to investigate the role of miRNAs in the EEC during EMT. The expression of SMAD3, SMAD5, and a panel of EMT markers was detected by Western blot and quantitative PCR.

**Results:**

The results of miRNA profiling in human EEC tissues and corresponding nontumorous endometriums demonstrated that miR-23a expression was down-regulated. Using bioinformatics, we identified SMAD3 or SMAD5 maybe as a predicted target of miR-23a. The results of luciferase reporter assay showed miR-23a directly targets and down-regulates human SMAD3 protein levels, not SMAD5 protein levels. Furthermore, overexpression of miR-23a in HEC-1-A cells increased E-cadherin expression and decreased the expression of vimentin and alpha smooth muscle actin, markers of mesenchymal cellular phenotype.

**Conclusions:**

Our data provide firm evidence of a role for miR-23a in the direct regulation of EMT through its targeting of SMAD3. Due to its ability to repress the EMT, miR-23a may be a novel target for EER therapeutic intervention.

**Electronic supplementary material:**

The online version of this article (doi:10.1186/s12935-016-0342-1) contains supplementary material, which is available to authorized users.

## Background

Endometrial cancer is the fourth most common malignancy of the female genital tract worldwide [1], and the major histological type of endometrial cancer is endometrial endometrioid adenocarcinoma (EEC) [2]. Although the integrated diagnosis and treatment provide significant insights into EEC, it still has some limitations including disease biology, morbidity and mortality. Therefore, a better understanding of the molecular mechanism of EEC maybe helpful to develop more effective therapies for the treatment of this disease.

Epithelial-to-mesenchymal transition (EMT) is a well-known mechanism for invasion (or metastasis) for many types of cancer [3]. It is a process during which tumor cells with epithelial characteristics lose their differentiated phenotypes and change their morphology to those characteristic of mesenchymal cells and migrate to the extracellular matrix [4]. During EMT, the epithelial markers of tumor cells were lost, such as E-cadherin and certain cytokeratins. Whereas, tumor cells gain mesenchymal markers such as vimentin and fibronectin [5]. EMT program plays important roles in promoting tumor cell invasion and chemoresistance of endometrioid adenocarcinoma cells via several transcription factors and related pathways, such as SNAIL (SNAI1), and SLUG (SNAI2) [6]. It was reported that SLUG expression serves as a prognostic factor in EEC [7]. In addition, one of the crucial signaling pathways of EMT in the pathogenesis of cancer is the transforming growth factor-beta/mothers against decapentaplegic homolog (TGF-β/SMAD) signaling pathway. The disturbances of the TGF-β pathway and overexpression or upregulation of SMAD4 expression were found to be important to the infiltration of the type I EEC [8]. However, loss of SMAD4 protein expression occurs infrequently in EEC [9]. SMAD3 is another member of the Smad proteins, which mediates the signals from the TGF-β superfamily ligands that regulate cell proliferation, differentiation and death [10]. Based on its essential role in TGF-β signaling pathway, SMAD3 is related with tumor growth in cancer development. It was reported upregulated SMAD3 promotes EMT and predicts poor prognosis in pancreatic ductal adenocarcinoma [11]. Therefore, EMT status maybe a molecular target in EEC.

A microRNA (miRNA) is a small non-coding RNA molecule (containing about 22 nucleotides) found in plants, animals, and some viruses, which functions in post-transcriptional regulation of gene expression [12]. So far, several studies showed the miRNA-dependent posttranscriptional regulation of genes associated with EEC development [13, 14]. Also EMT can be controlled by a specific set of miRNA. In the present study, due to the important role of EMT in EEC, we analyze all miRNAs expression profile of EEC in order to search for EMT-related miRNAs and investigate their role in EEC. Using an unbiased miRNA screen, we identified miR-23a as an down-regulated miRNA in EC. Through in silico prediction and luciferase reporterassay, we found that SMAD3, may be one of miR-23a target genes in EEC. Thus, we demonstrate that miR-23a might be as an EMT-related miRNA in EEC by targeting SMAD3.

## Methods

### Samples

Biospecimens of endometrioid adenocarcinoma and corresponding nontumorous endometriums from 30 patients diagnosed with endometrioid adenocarcinoma were used after institutional review board-approved consents. All the patients were treated at Changning Maternity and Infant Health Hospital (Shanghai, China) in 2009–2011. Inclusion criteria were all women greater than 45 years of age with histological diagnosis consistent with EEC. Among these cases, 25 were postmenopausal. The pieces of tumor tissue and nontumorous endometrium tissues were carefully selected and snap frozen in liquid nitrogen, followed by storage at −80 °C.

### RNA isolation, quantitative reverse transcriptase polymerase chain reaction and miRNA expression profiling and validation

For E-cadherin, Vimentin and α-SMA mRNA expression, total RNA was isolated using the RNeasy^®^ Mini Kit (Qiagen, Hilden, Germany) and transcribed to cDNA using the SuperScript^®^ Reverse Transcriptase (Invitrogen, Carlsbad, CA, USA). Primer sequences used in this study are provided in Additional file 1: Table S1. Data were normalized using human GAPDH housekeeping gene and analyzed by comparing the 2^−ΔCt^ values of the normalized data.

For miRNA expression profiling, total RNA was isolated with a mirVana™ miRNA Isolation Kit (Ambion, Austin, TX, USA). miRNA microarray analysis, including labeling, hybridization, scanning, normalization and data analysis, was performed by CloudSeq Bio-tech, Shanghai, China, on a miRCURY LNA™ microRNA Array Kit v.19.0 (Exiqon, Vedbaek, Denmark). The microRNA profiling contains 3100 capture probes cover human, mouse and rat microRNAs.

All specific primers for miRNA expression were designed and synthesized by Guangzhou RiboBio Co Ltd, Guangzhou, China, using the mirVana™ qRT-PCR Primer Sets. The levels of an endogenous control, U6 (RiboBio, Guangzhou, China), were used to normalize the expression levels of each miRNA. All reactions were performed in triplicate and included controls without a template for each miRNA. The fold change in miRNA expression was calculated using the comparative CT method.

### Cell culture

HEC-1-A cells (human endometrial carcinoma cells-1-A) are human endometrium adenocarcinoma cell line and were obtained from American Type Culture Collection (Manassas, VA, USA) and were cultured in McCoy’s 5A modified medium supplemented with 10 % fetal bovine serum and 1 % penicillin/streptomycin (Sigma-Aldrich, St Louis, MO, USA). HEC-1-A cells are used as an accepted model to study endometrial cancer [15]. MiR-23a mimic, inhibitor, agomir or antagomir were purchased from RiboBio Inc (GuangZhou, China). Human recombinant TGFβ1 was obtained from R & D Systems Inc (Minneapolis, MN, USA).

### MicroRNA target gene prediction

In silico programs, a computational approach was used to predict the miRNA target genes with the following three different miRNA target prediction algorithms: PicTar, miRanda, and TargetScan. The potential binding sites in the messenger RNA 3′ according to specific base-pairing rules were identified, and second, implementation of cross-species conservation requirements was performed.

### Luciferase activity assay

Human SMAD3 or SMAD5 3′-UTR, containing the putative target site for miR-23a, was amplified from genomic DNA by PCR amplification and inserted into the pmiR-REPORT™ (RiboBio). The mutation from a site of perfect complementarity was also generated by the QuikChange II^®^ Site-Directed Mutagenesis Kit (Stratagene, La Jolla, CA, USA) and by following the manufacturer’s instructions. The HEC-1-A cells were transiently transfected with wild-type or mutant reporter plasmid. Luciferase activity was measured 24 h after transfection, as described previously. Three independent experiments were performed in triplicate.

### Immunoblotting

Samples were homogenized in 100 μL ice-cold radioimmunoprecipitation assay lysis buffer (Beyotime Institute of Biotechnology, Haimen, China) supplemented with a proteinase inhibitor cocktail. The blots were probed with the primary antibodies for E-cadherin (ab76055, Abcam, Cambridge, MA, USA), Vimentin (ab92547, Abcam), α-SMA (ab5694, Abcam), SMAD3 (ab40854, Abcam) and SMAD5 (ab40771, Abcam).

### Statistical analyses

**Fig. 1:**
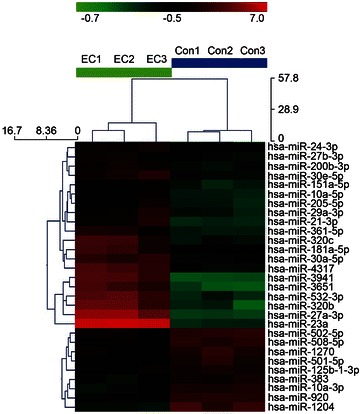
miRNA profiles in EER tissues and corresponding nontumorous endometriums. Hierarchical clustering was performed with the normalized miRNA data (greater than twofold change). A total of 178 miRNAs were identified as having significantly altered expression between the EER tissues and corresponding nontumorous endometriums. This figure depicts some of the 178 regulated miRNAs. *Rows* miRNA; *Column* EEC tissues (EC1, EC2, and EC3) and corresponding nontumorous endometriums (Con1, Con2, and Con3). For each miRNA, the *red color* indicates genes with high expression, and the *green color* denotes genes with low expression

The results are expressed as the mean ± standard deviation (SD). For the results shown in Fig. 1, the difference was calculated using an unpaired t test. For the other results, statistical analyses were performed using a one-way analysis of variance by comparing the groups using the Student–Newman–Keuls test. The least significant difference procedure was performed with GraphPad Prism software 5.0 (GraphPad Software, Inc., San Diego, CA). A P value <0.05 was considered to be statistically significant.

## Results and discussion

### Differential miRNA expression between endometrioid adenocarcinoma and adjacent nontumorous endometrium

The samples were pooled into six groups (con1, con2, con3, EC1, EC2, and EC3. Each group comprised ten samples. Based on the results of a *t* test, a Volcano plot filtering was performed between the two groups. The threshold we used to screen up- or down-regulated miRNAs was a fold change ≥2.0 and a P value ≤0.05. A total of 178 miRNAs were found to be differentially expressed, and only 37 miRNAs passed the Volcano plot filtering screen at the significance level (P < 0.05, false discovery rate <0.05). Among these miRNAs, 20 were found to be down-regulated less than 0.5-fold, and 17 miRNAs were found to be up-regulated more than 2.0-fold in the EEC samples compared to the adjacent nontumorous endometrium samples (Fig. 1). The expression of the most prominent miRNA, miR-23a was down-regulated almost tenfold.

**Fig. 2:**
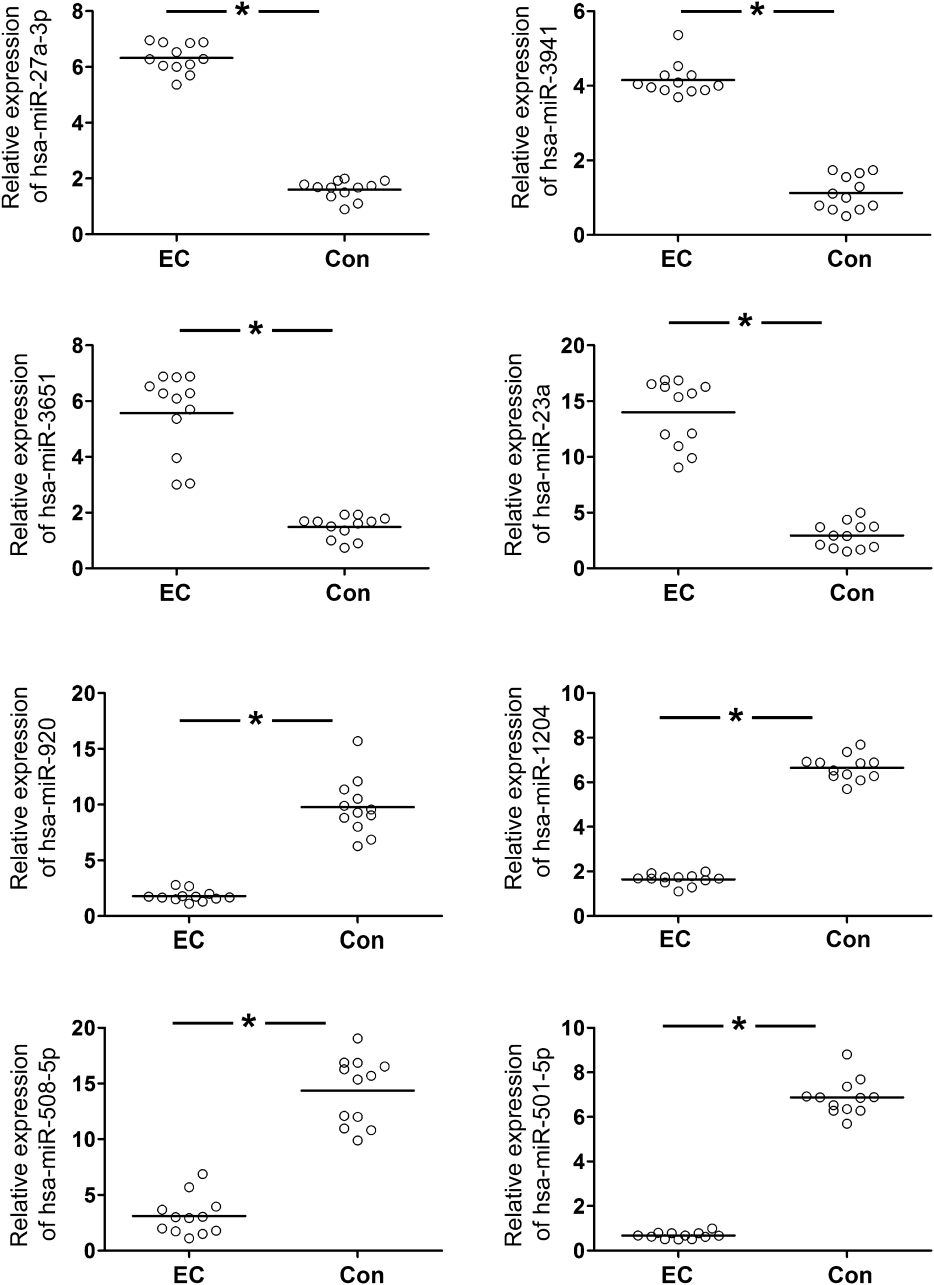
Validation of select microarray data by qRT-PCR. We also collected another 12 EEC and adjacent nontumorous endometrium samples and were used for qPCR detection. Hsa-miR-23a, hsa-miR-3941, hsa-miR-27a-3p and hsa-miR-3651 were the most significantly down-regulated miRNAs, whereas hsa-miR-920, hsa-miR-1204, hsa-miR-508-5p and hsa-miR-501-5p were the most significantly up-regulated miRNAs (n = 12 per group). The relative amount of each miRNA was normalized to the U6 snRNA. Significant differences between the EEC tissues and adjacent nontumorous endometrium samples are indicated by an asterisk (*P < 0.05)

We also collected another 12 EEC and adjacent nontumorous endometrium samples and were used for qPCR detection. The qPCR assay was used to validate the eight selected miRNAs. Hsa-miR-23a, hsa-miR-3941, hsa-miR-27a-3p and hsa-miR-3651 were the most significantly down-regulated miRNAs, whereas hsa-miR-920, hsa-miR-1204, hsa-miR-508-5p and hsa-miR-501-5p were the most significantly up-regulated miRNAs (n = 12 per group) (Fig. 2). These data were consistent with the microarray results. One of the down-regulated miRNAs, miR-23a, merited further investigation because it was predicted to target SMAD3 or SMAD5, which plays an important role in EMT. We wanted to investigate if miR-23a plays a role in EMT associated to EEC. We didn’t find any known correlation between miR-23a and the clinico-pathological features of EEC through the literature search.

### MiR-23a directly targets and down-regulates human SMAD3 protein levels, not SMAD5

**Fig. 3:**
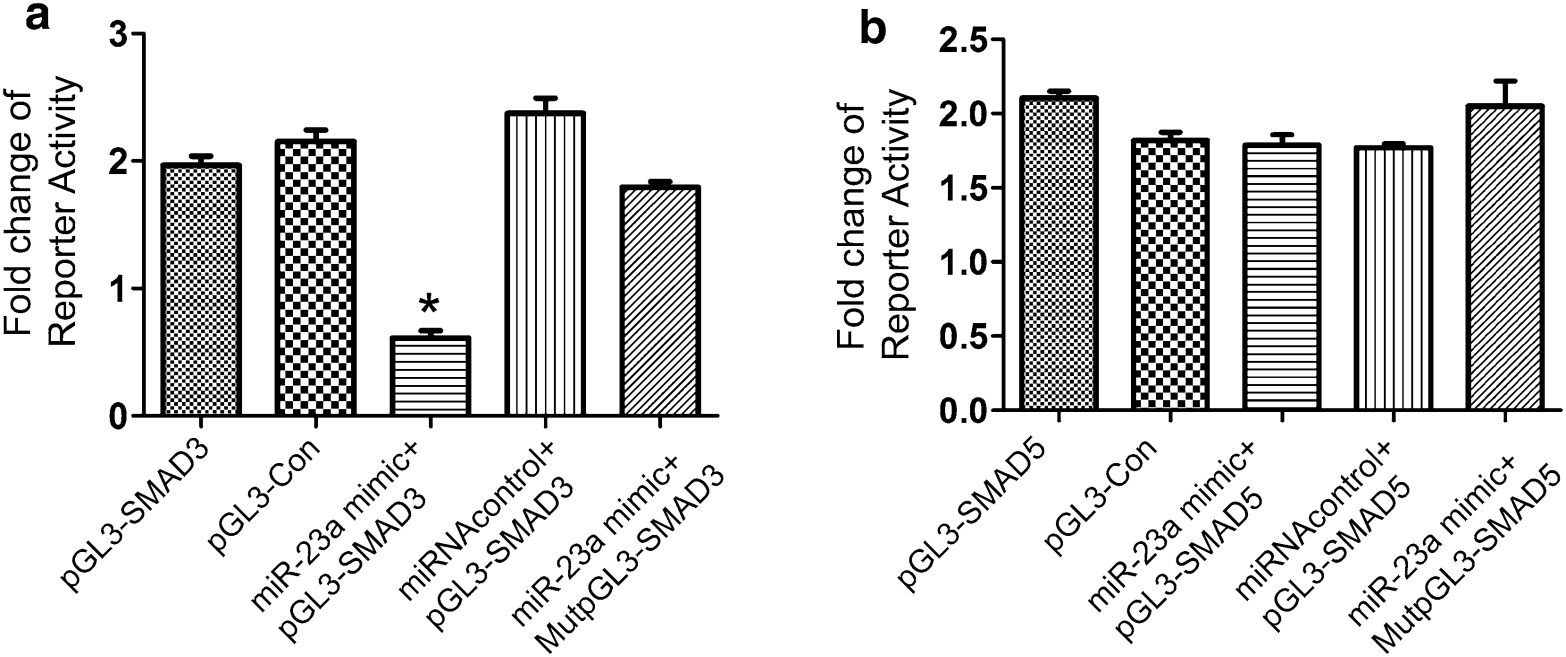
Results of dual luciferase reporter assay in HEC-1-A cells. From the bioinformatics database, miR-23a was predicted to target the SMAD3 (**a**) or SMAD5 (**b**) gene at a site in the 3′-UTR. To test the possibility of a direct link between miR-23a and human SMAD3 or SMAD5, we performed a dual luciferase reporter assay in HEC-1-A cells. A significant decrease in relative luciferase activity was observed when pGL3-SMAD3-3′-UTR was cotransfected with a miR-23a mimic. Whereas, we didn’t find any significant decrease in relative luciferase activity when pGL3-SMAD5-3′-UTR was cotransfected with a miR-23a mimic. In the presence of the miR-23a mimic, expression of the renillaluciferase reporter was repressed 2.9-fold compared with the vector-only control. Significantly, partial deletion of the perfectly complementary sites in the 3′-UTR of SMAD3 abolished the suppressive effect due to the disruption of the interaction between miR-23a and SMAD3. Triplicate assays were performed for each sample (n = 6 per group) (*P < 0.05)

In silico programs, including PicTar, miRanda, and TargetScan, we found SMAD3 and SMAD5 maybe the target genes for miR-23a with a binding motif. We found the TGF-β/SMAD signaling pathway was significantly enriched among the predicted targets of miR-23a (P = 0.032). From this bioinformatics database, miR-23a was predicted to target the SMAD3 or SMAD5 gene at a site in the 3′-UTR. To test the possibility of a direct link between miR-23a and human SMAD3 or SMAD5, we performed a dual luciferase reporter assay in HEC-1-A cells. A significant decrease in relative luciferase activity was observed when pGL3-SMAD3-3′-UTR was cotransfected with a miR-23a mimic. Whereas, we didn’t find any significant decrease in relative luciferase activity when pGL3-SMAD5-3′-UTR was cotransfected with a miR-23a mimic. In the presence of the miR-23a mimic, expression of the renillaluciferase reporter was repressed 2.9-fold compared with the vector-only control. Significantly, partial deletion of the perfectly complementary sites in the 3′-UTR of SMAD3 abolished the suppressive effect due to the disruption of the interaction between miR-23a and SMAD3 (Fig. 3).

**Fig. 4:**
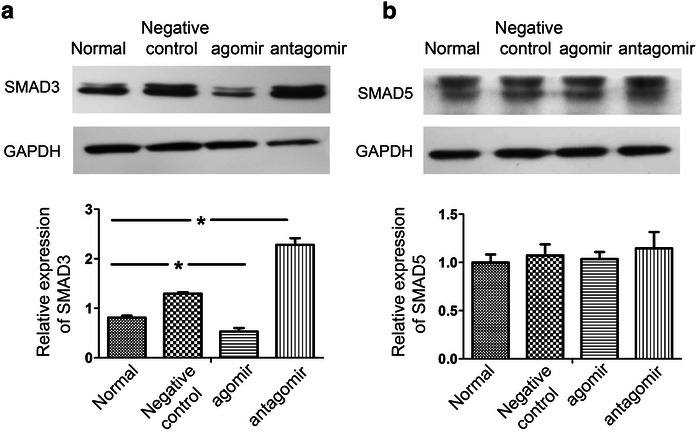
SMAD3 protein levels are down-regulated by miR-23a. **a** SMAD3 protein expression was decreased by treatment with the miR-23a agomir but increased by treatment with a miR-23a antagomir in HEC-1-A cells (*top* data from the gels; *bottom* normalization to GAPDH) (n = 6 per group) (*P < 0.05). **b** SMAD5 protein expression was decreased by treatment with the miR-23a agomir but increased by treatment with a miR-23a antagomir in HEC-1-A cells (*top* data from the gels; *bottom* normalization to GAPDH). SMAD5 protein level was not significantly influenced by the overexpression or inhibition of miR-23a (n = 12 per group)

Furthermore, SMAD3 protein expression was decreased by treatment with the miR-23a agomir but increased by treatment with a miR-23a antagomir in HEC-1-A cells (Fig. 4). However, SMAD3 mRNA levels were not significantly influenced by the overexpression or inhibition of miR-23a (data not shown), suggesting that SMAD3 expression was primarily inhibited by miR-23a at the translational level. SMAD5 protein expression was also detected by Western blot and its protein level was not significantly influenced by the overexpression or inhibition of miR-23a. Together, these results confirmed that SMAD3, not SMAD5 is a direct target of miR-23a and is regulated by miR-23a.

### Up-regulation of miR-23a repressed the TGF-β induced EMT in HEC-1-A cells

**Fig. 5:**
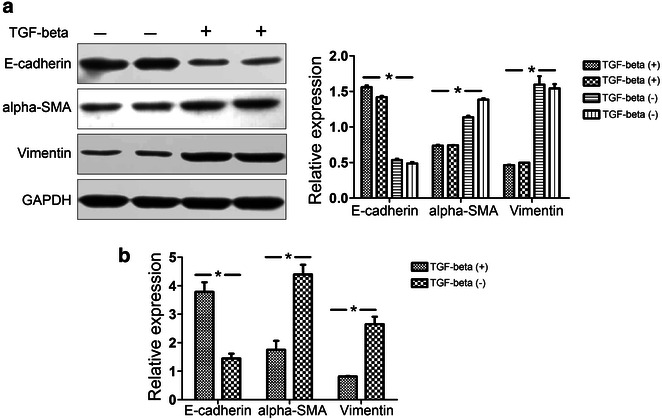
The protein and mRNA levels of E-cadherin, α-SMA, and vimentin were quantified by Western blot and qPCR analyses on TGF-β1 induced EMT in HEC-1-A cells. **a** The results of Western blot. (*left* data from the gels; *right* normalization to GAPDH) (n = 6 per group) (*P < 0.05). **b** The results of qPCR (n = 6 per group) (*P < 0.05)

In the present study, we used TGF-β1 to induce EMT in HEC-1-A cells. The markers of EMT, E-cadherin, α-SMA, and vimentin, were detected. Within 3 days of culture, HEC-1-A cells were starved for a further 24 h and then treated with 10 ng/mL TGF-β1. After 24 h of TGF-β1 stimulation, the protein and mRNA levels of E-cadherin, α-SMA, and vimentin were quantified by Western blot and qPCR analyses (Fig. 5). Expression of E-cadherin was highest when within the cells with no TGF-β1 treatment and decreased in cells treated with TGF-β1 by TGF-β1 treatment. In contrast, expression of vimentin and α-SMA increased over the duration of the TGF-β1 treatment.

**Fig. 6:**
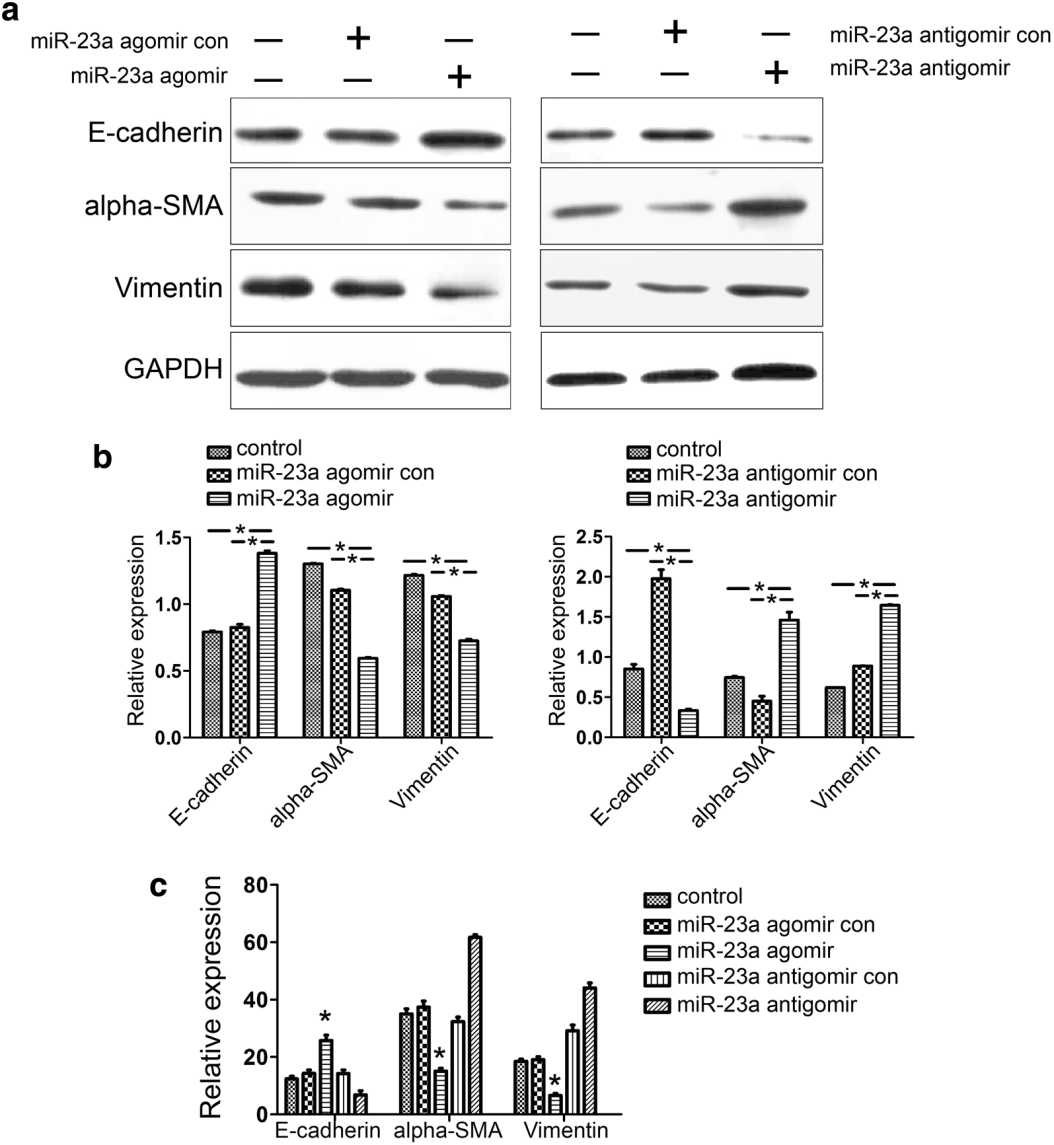
Expression of EMT markers when miR-23a is overexpressed in HEC-1-A cells. This experiment was conducted using treatment with 10 ng/mL TGFβ1 for 24 h, and the cells were then treated with the miR-23a agomir or antagomir investigate the effect of miR-23a on the EEC EMT. Non-treatment of TGFβ1 was used as the control group, and each group contained three samples. **a**, **b** Protein expression levels of the EMT markers. E-cadherin protein expression was increased when the HEC-1-A cells were treated with the miR-23a agomir but decreased when the cells were treated with the miR-23a antagomir. In contrast, the expression of both α-SMA and vimentin was decreased when the HEC-1-A cells were treated with the miR-23a agomir but increased when the cells were treated with the miR-23a antagomir. *Top* the gels (**a**); *bottom* the normalization graphs (**b**). **c** Up-regulation of miR-23a repressed the mRNA expression of EMT markers. The expression of mRNAs encoding α-SMA and vimentin was reduced in HEC-1-A cells treated with the miR-23a agomir but increased in cells treated with the miR-23a antagomir. However, the expression of E-cadherin mRNA was increased by the miR-23a agomir and decreased by the antagomir (n = 6 per group) (*P < 0.05)

To further investigate the role of miR-23a in the development of EEC by its ability to repress EMT, miR-23a agomir or antagomir were used to treat the TGF-β induced EMT in HEC-1-A cells. The results shown in Fig. 6 illustrate that E-cadherin protein expression increased when the HEC-1-A cells were treated with the miR-23a agomir but decreased when the HEC-1-A cells were treated with the miR-23a antagomir. In contrast, the protein expression of both α-SMA and vimentin decreased when the HEC-1-A cells were treated with the miR-23a agomir but increased when the cells were treated with the miR-23a antagomir. Similarly, E-cadherin mRNA levels were increased by the miR-23a agomir but decreased by the miR-23a antagomir, while α-SMA and vimentin mRNA levels were decreased in HCE-1-A cells treated with the miR-23a agomir but increased in HCE-1-A cells treated with the miR-23a antagomir. These results clearly show the up-regulation of miR-23a repressed the TGF-β induced EMT biomarkers in HEC-1-A cells.

## Discussion

In this study, we demonstrated differently expressed miRNA in EEC and identified potential miRNA modulator of EMT in endometrial endometrioid adenocarcinoma. We identified potential miRNA modulators of EMT in endometrial endometrioid adenocarcinoma. MiR-23a was selected for further analysis because it was down-regulated approximately tenfold. Because the target genes of miR-23a were SMAD3 or SMAD5, which were the key regulator of EMT, miR-23a was a potential regulator of EMT in EEC.

MiR-23a has previously been shown to be expressed in neuronal precursor cells and hindered the retinoic-acid-induced neuronal differentiation of NT2 cells by targeting *Hes1* (hairy and enhancer of split-1) gene [16]. Additionally, miR-23a is required to restrict endocardial cushion formation and extracellular hyaluronic acid production [17]. It was reported miR-23 is enriched in endothelial cells and highly vascularized tissues. Inhibition of miR-23 represses angiogenesis in vitro and postnatal retinal vascular development in vivo. Moreover, miR-23 is required for pathological angiogenesis in a laser-induced choroidal neovascularization mouse model by promoting angiogenic signaling through targeting Sprouty2 and Sema6A proteins [18]. MiR-23a had also been identified in a comparison between EEC and nontumor and it was not reported to be the most differentially expressed [19]. Recently, the expression of miR-23a was decreased in osteosarcoma cells and as a tumor suppressor in osteosarcoma [20]. Several targets of miR-23a were previously validated in other tumor cell types, including RUNX2 and CXCL12 in osteosarcoma. Downregulation of miR-23a suppresses prostate cancer metastasis by targeting the PAK6-LIMK1 signaling pathway [21]. However, there has no report about targets of miR-23a in EMT.

In the present study, we found that miR-23a was significantly down-regulated in human EEC samples. Based on the significant down-regulation of miR-23a in human EEC, we undertook detailed mechanistic studies. We showed that miR-23a targets the 3′-UTR of SMAD3 and inhibits TGF-β induced EMT in HEC-1-A cells. SMAD3 was found to promote EMT and predict prognosis in pancreatic ductal adenocarcinoma [11]. Our data suggest the regulation of miR-23a may be SMAD3-dependent because SMAD3 expression was significantly suppressed when miR-23a was overexpressed. Moreover, the expression of SMAD5 was unaffected in the presence of either miR-23a mimic or inhibitor. This result indicated that SMAD3, rather than SMAD5, is a specific target gene of miR-23a in the regulation of EMT in EEC. SMAD3 functions as a transcriptional modulator, binding the TRE (TPA responsive element) in the promoter region of many genes that are regulated by TGF-β. The role of SMAD3 in the regulation of genes important for cell fate, such as differentiation, growth and death, implies that an alteration in its activity or repressing of its activity can lead to the formation or development of cancer. SMAD5 belongs to the SMAD family of proteins, which belong to the TGF-β superfamily of modulators. SMAD5 is involved in cell signaling and modulates signals of bone morphogenetic proteins (BMP’s). The binding of ligands causes the oligomerization and phosphorylation of the SMAD5 protein. It may play a role in the pathway where TGF-β is an inhibitor of hematopoietic progenitor cells [22].

We next investigated whether EMT formation could be successfully attenuated by regulating miR-23a in HEC-1-A cells. Common established markers of the EMT are the loss of E-cadherin expression and the gain of a-SMA and vimentin expression. In the present study, we observed that these EMT-associated markers were perturbed by miR-23a overexpression or knockdown. The results revealed that EEC-associated EMT was inhibited, since the expression of both the transdifferentiation marker a-SMA and mesenchymal marker vimentin were inhibited, and the epithelial marker E-cadherin was restored when HEC-1-A cells were treated with the miR-23a agomir. However, when the HEC-1-A cells were treated with the miR-23a antagomir, changes in the expression level of these three EMT markers were reversed. TGF-β/SMAD3 signaling is critical in the induction of EMT [23, 24]. In addition, gene ablation of SMAD3 in mammary gland epithelial cells prevents the EMT [25].

## Conclusion

In conclusion, we have identified a molecular mechanism to be involved in EMT during the development of EEC involving miR-23a and SMAD3. The potential therapeutic role of miRNAs in the prevention of EEC remains a major clinical challenge. The presented evidence underscores the importance of miR-23a as a novel target for therapeutic intervention and indicates that this miRNA merits further investigation as a promising gene therapy target for the treatment of endometrial endometrioid adenocarcinoma.

## Additional file

**Additional file 1: Table S1.** Primers used in qPCR.

## Abbreviations

EEC: endometrial endometrioid adenocarcinoma
EMT: epithelial-to-mesenchymal transition
miRNA, miR: microRNA
