# Supplementary material for: MicroRNA-23a regulates epithelial-to-mesenchymal transition in endometrial endometrioid adenocarcinoma by targeting SMAD3
Source: Cancer Cell Int. 2016 Sep 5;16:67. doi: 10.1186/s12935-016-0342-1 (PMC5011925; doi:10.1186/s12935-016-0342-1)
Supplement: Supplementary file 1 — Additional file 1: Table S1. Primers used in qPCR. [file 12935_2016_342_MOESM1_ESM.docx]

**Table S1. Primers used in qPCR**

| **Gene Name** | **Primer Sequences** | **Product Size (bp)** | |
| --- | --- | --- | --- |
| ***E-cadherin*** | F: cggacgatgatgtgaacacc | | 213 |
| (NM_004360) | R: aacatcgcagtctccaagga | |  |
| ***Vimentin***  (NM_003380) | F: tgcaggctcagattcaggaa  R: ctccggtactcagtggactc | | 229 |
| ***α-SMA*** | R: acccagcaccatgaagatca | | 157 |
| (NM_001141945) | R: tttgcggtggacaatggaag | |  |
